# Care pathways following disclosure of adolescent non-suicidal self-injury: a dyadic qualitative study

**DOI:** 10.3389/fpsyt.2026.1934223

**Published:** 2026-09-30

**Authors:** Jinlin Fang, Dongqin Xu, Chao Qian, Xiaofang Qian, Yan Xu

**Affiliations:** Shaoxing Seventh People’s Hospital (Affiliated Mental Health Center, Shaoxing University), Shaoxing, China

**Keywords:** adolescent, caregivers, continuity of care, help-seeking, non-suicidal self-injury, psychiatric services, qualitative research

## Abstract

**Introduction:**

Adolescents with non-suicidal self-injury (NSSI) often move between family, school, and mental health services after disclosure, but the care pathways shaping psychiatric service engagement and continuity remain poorly understood.

**Methods:**

We conducted a dyadic qualitative study using semi-structured interviews with 20 adolescents (14–17 years) with a history of NSSI and 20 caregivers in Shaoxing, China. Reflexive thematic analysis was combined with socioecological journey mapping to reconstruct post-disclosure care trajectories across individual, family, school, and health-system contexts.

**Results:**

Analysis identified a six-stage care pathway from accumulated distress and concealed NSSI to discovery, multi-system service entry, treatment engagement, and continuing care needs. The key transition was the point at which NSSI became visible to others. Participants described post-disclosure responses as important contexts associated with more stabilizing or fragmented care trajectories. Adolescents emphasized shame, exposure, and uncertainty, whereas caregivers described fear, guilt, and difficulty identifying appropriate help. Continuity depended on emotional safety, caregiver reframing, school referral, clear service navigation, and follow-up planning.

**Discussion:**

Disclosure or discovery of adolescent NSSI is a clinically important transition that shapes subsequent psychiatric service engagement and continuity of care. Coordinated responses across families, schools, and mental health services may help transform disclosure from a crisis event into a pathway toward sustained support.

## Introduction

Non-suicidal self-injury (NSSI) refers to deliberate self-inflicted damage to body tissue without suicidal intent. It is clinically important in adolescence because it is associated with emotional dysregulation, distress, impaired family communication, and later risk, even when suicidal intent is absent. Prior work has emphasized the affect-regulation and self-punishment functions of self-injury, while clinical guidance stresses careful psychosocial assessment, risk management, and prevention of recurrence (1–4).

A persistent challenge is that adolescents rarely enter care through a simple, linear pathway. NSSI may remain hidden for months, and disclosure can occur through peers, parents, school staff, emergency services, or outpatient contacts. Reviews of NSSI disclosure and adolescent help-seeking show that stigma, embarrassment, limited knowledge, distrust of services, and logistical barriers can delay professional support (5–8). Parents also experience shock, fear, and uncertainty after discovering adolescent self-harm (9). Although this evidence is derived from the broader self-harm literature rather than NSSI-specific studies, it provides relevant contextual insight into caregiver responses following adolescent NSSI disclosure.

Most clinical discussions focus on assessment and treatment after service contact. Less attention has been given to the transitional journey from concealed distress to sustained care across home, school and health systems. A socioecological lens is useful because it treats adolescent behavior, family response, school detection, and service organization as interacting levels rather than separate causes (10). Journey mapping adds a temporal dimension by following participants across stages, touchpoints, barriers, and stabilizing supports.

This study used paired adolescent-caregiver qualitative materials to map the care journey after adolescent NSSI. The objectives were to describe sample and pathway characteristics, identify common journey stages, synthesize socioecological barriers and facilitators, and generate a dyadic model of care pathway stability and instability.

To our knowledge, few studies have reconstructed adolescent NSSI care trajectories using paired adolescent-caregiver data and a socioecological journey-mapping framework. This study therefore contributes a process-oriented model of disclosure, service navigation, and continuity of care.

## Materials and methods

### Participants and recruitment

Participants were recruited between August 2025 and May 2026 from adolescent psychiatric outpatient services and collaborating secondary schools in Shaoxing, China. For recruitment and eligibility assessment, non-suicidal self-injury (NSSI) was operationalized as deliberate, self-inflicted damage to body tissue without suicidal intent. Eligible participants were adolescents aged 14–17 years with a documented history of NSSI and one corresponding caregiver involved in the adolescent’s daily care. Caregivers were included when they were able to provide accounts of the adolescent’s post-disclosure care experiences. The specific relationship between caregivers and adolescents (e.g., mother, father, or other caregiver) and other caregiver demographic characteristics were not systematically collected, because the study focused on relational processes and care pathways rather than caregiver-level demographic comparisons. Therefore, caregiver relationship information was not available for retrospective reporting. The exact distribution of recruitment sources (i.e., the number of dyads recruited through psychiatric outpatient services versus collaborating secondary schools) was not systematically recorded and therefore cannot be retrospectively reconstructed.

Potential participants were initially identified by treating psychiatrists at the adolescent psychiatric outpatient services or by school mental health staff and subsequently approached by the research team.

A documented history of NSSI was established based on clinical records from treating psychiatrists. For adolescents identified through collaborating secondary schools, NSSI status was confirmed through clinical assessment before enrolment. The diagnosis of NSSI and the absence of suicidal intent were determined by treating psychiatrists during routine clinical assessment based on established clinical criteria. No minimum frequency or recency threshold for NSSI was applied; adolescents were eligible if they had a documented history of NSSI. Adolescents with a history of suicide attempts, acute suicide risk, or those unable to participate meaningfully in an interview because of communication or cognitive difficulties were excluded. Interviews focused specifically on participants’ experiences of NSSI rather than suicidal self-harm.

Purposive sampling was used to ensure variation in age, sex, school grade, and help-seeking experience. The final sample comprised 20 adolescents and 20 caregivers.

### Data collection

Semi-structured interviews were conducted separately with adolescents and caregivers by trained researchers to minimize mutual influence and allow independent accounts of care experiences. Interviews were undertaken in private consultation rooms within the hospital or in quiet rooms provided by collaborating schools. Each interview lasted approximately 46–58 minutes. No repeat interviews were conducted. Eight eligible families declined participation because of time constraints or reluctance to discuss self-injury experiences.

The interview guide was developed from previous literature on NSSI disclosure, adolescent help-seeking, family response, and mental health service utilization. Following pilot testing and team discussion, the guide was refined to improve clarity and sensitivity. Interview topics included NSSI experiences, disclosure processes, family and school responses, help-seeking pathways, treatment engagement, and ongoing care needs.

All interviews were conducted in Mandarin Chinese, audio-recorded with consent, transcribed verbatim, and anonymized before analysis. Field notes were completed after each interview. Data sources also included demographic information, coding records, analytic memos, thematic-development logs, and journey-mapping matrices. Transcripts were not routinely returned to participants because of the sensitive nature of the topic and concerns regarding participant burden. Data were managed and coded using Microsoft Excel. Quotations included in the manuscript were translated from Chinese into English by a bilingual researcher and independently checked by a second bilingual researcher to ensure conceptual equivalence.

### Research team and reflexivity

The interviews were conducted by a psychiatric nurse researcher and a child psychiatrist, both of whom had prior experience in adolescent mental health qualitative research. No prior relationship existed between researchers and participants before recruitment. The study was conducted from a contextualist epistemological orientation, and analysis was approached as an interpretive and reflexive process rather than as a search for coding consensus or coding reliability. Reflexive journals were maintained throughout data collection and analysis to document researchers’ assumptions, clinical backgrounds, positionality, and analytic decisions. Regular team discussions were used to examine how professional experience might shape interpretation of adolescents’ and caregivers’ accounts, to consider alternative readings, and to refine analytic questions. The involvement of multiple researchers therefore supported reflexive interrogation and analytic richness rather than inter-coder agreement.

### Data analysis

Data collection and analysis were conducted iteratively. Interpretive sufficiency was considered reached when additional interviews no longer substantially elaborated the developing conceptual categories or pathway transitions across three consecutive interviews. This decision was documented in analytic memos and discussed within the research team as part of reflexive assessment of the developing analysis, rather than as a test of coding saturation or coding reliability.

Analysis proceeded through a sequential interpretive workflow (11, 12). First, participant and pathway characteristics were summarized descriptively. Second, interview transcripts were read repeatedly, and meaning units relevant to NSSI disclosure, family response, school contact, service entry, treatment engagement, and continuing care needs were identified and organized chronologically across the NSSI care trajectory. Third, all transcripts were initially coded by one researcher (JLF), who conducted repeated readings, generated initial codes, and developed preliminary interpretations. Other members of the research team participated in reflexive discussions of emerging codes and themes, providing alternative perspectives and contributing to the refinement of interpretations. These discussions were not intended to achieve coding consensus or assess inter-coder reliability, but rather to support reflexive engagement and analytic depth. Coding decisions were documented through reflexive memo-writing, analytic records, and thematic-development logs. Codes were treated as flexible interpretive tools rather than units for coding reliability, consistent with a reflexive thematic analysis approach.

The dyadic analytical strategy involved both within-dyad and across-dyad comparison. Within each adolescent-caregiver dyad, the adolescent and caregiver interviews were examined together to compare how each member described NSSI disclosure or discovery, family response, school or clinical contact, treatment engagement, and continuing care needs. Concordant interpretations were identified when adolescent and caregiver accounts described similar pathway transitions, care touchpoints, barriers, or facilitators. Discordant interpretations were identified when adolescent and caregiver accounts differed in their interpretation of disclosure, caregiver response, service navigation, emotional meaning, or continuing care needs. Concordant and discordant interpretations were documented within the dyadic analytic matrices and analytic memos.

Journey-mapping matrices were developed by JLF during the analytic process to organize dyadic information chronologically and socioecologically. These matrices were subsequently discussed with the research team to support reflexive interpretation and refinement of emerging themes. For each dyad, matrix entries captured the sequence of care stages, relevant family, school, and health-service touchpoints, perceived barriers and facilitators, and adolescent-caregiver similarities or differences in interpretation (13). Within-dyad comparison contributed to theme development by identifying relational patterns, especially points at which adolescents’ and caregivers’ accounts converged or diverged around disclosure, help-seeking, and continuity of care.

Fourth, inductively developed codes were synthesized into candidate themes describing recurring care processes, barriers, facilitators, and transition points. These themes were then used to construct six empirically grounded stages of the NSSI care pathway: accumulated distress, concealment and delayed disclosure, discovery and visibility transition, fragmented multi-system service entry, treatment engagement and family adaptation, and non-linear recovery with continuing care needs. These stages reflected recurring temporal and experiential patterns in participants’ accounts. Journey-mapping matrices were then used to arrange the stages chronologically and to link each stage with relevant touchpoints, barriers, facilitators, and individual, family, school, or health-system levels.

Across-dyad comparison was used to examine whether similar within-dyad patterns recurred across families and how these patterns clustered around shared care transitions. These comparisons informed the synthesis of codes into higher-order themes and the mapping of themes onto a six-stage socioecological care pathway. The final pathway was developed by integrating individual accounts, within-dyad concordance and discordance, and cross-dyad similarities and contrasts across individual, family, school, and health-system domains.

The six-stage journey map was interpreted through a socioecological lens to examine how adolescent experiences, caregiver responses, school processes, and health-service contacts interacted across time. This socioecological interpretation was an author-developed conceptual synthesis built from the inductive themes and journey-mapping matrices. Finally, the bifurcation model was developed as a conceptual interpretation of the observed divergence in post-disclosure trajectories. The inductive finding was that participants described different pathway patterns after NSSI became visible, including more stabilizing trajectories when responses were validating and coordinated, and more fragmented trajectories when responses were punitive, delayed, or unclear. The authors’ conceptual contribution was to interpret this divergence as a socioecological bifurcation process, in which disclosure or discovery functioned as a transition point after which care pathways could become stabilized or fragmented in relation to cross-level responses. For figure preparation, **Figures 1**, **2** were treated as visual syntheses rather than quantitative displays: no numerical values, scale scores, or frequency counts were assigned to the emotional-pattern strip in **Figure 1**, and **Figure 2** visually separates empirical pathway themes from contextual moderators informed by participant accounts and prior literature.

**Figure 1:**
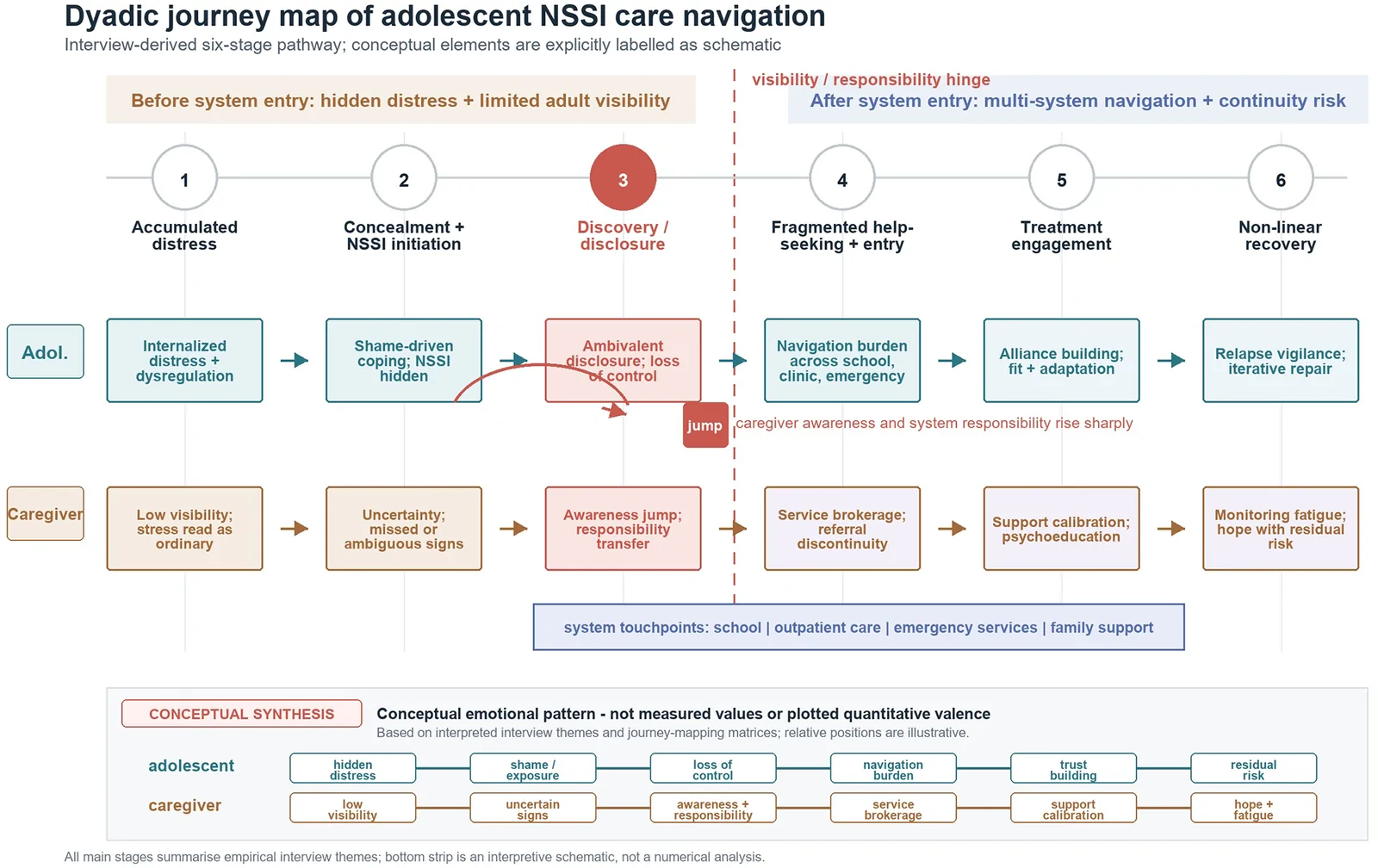
Dyadic journey map of adolescent NSSI care navigation. The upper track represents the adolescent pathway, and the lower track represents the caregiver pathway. The visibility-responsibility hinge marks the critical transition from concealed distress to shared multi-system care engagement, where responsibility for care shifts across adolescent, family, school, and healthcare systems. The emotional-pattern strip represents a conceptual synthesis of interpreted adolescent and caregiver experiences and does not represent quantitative measurement or scoring.

**Figure 2:**
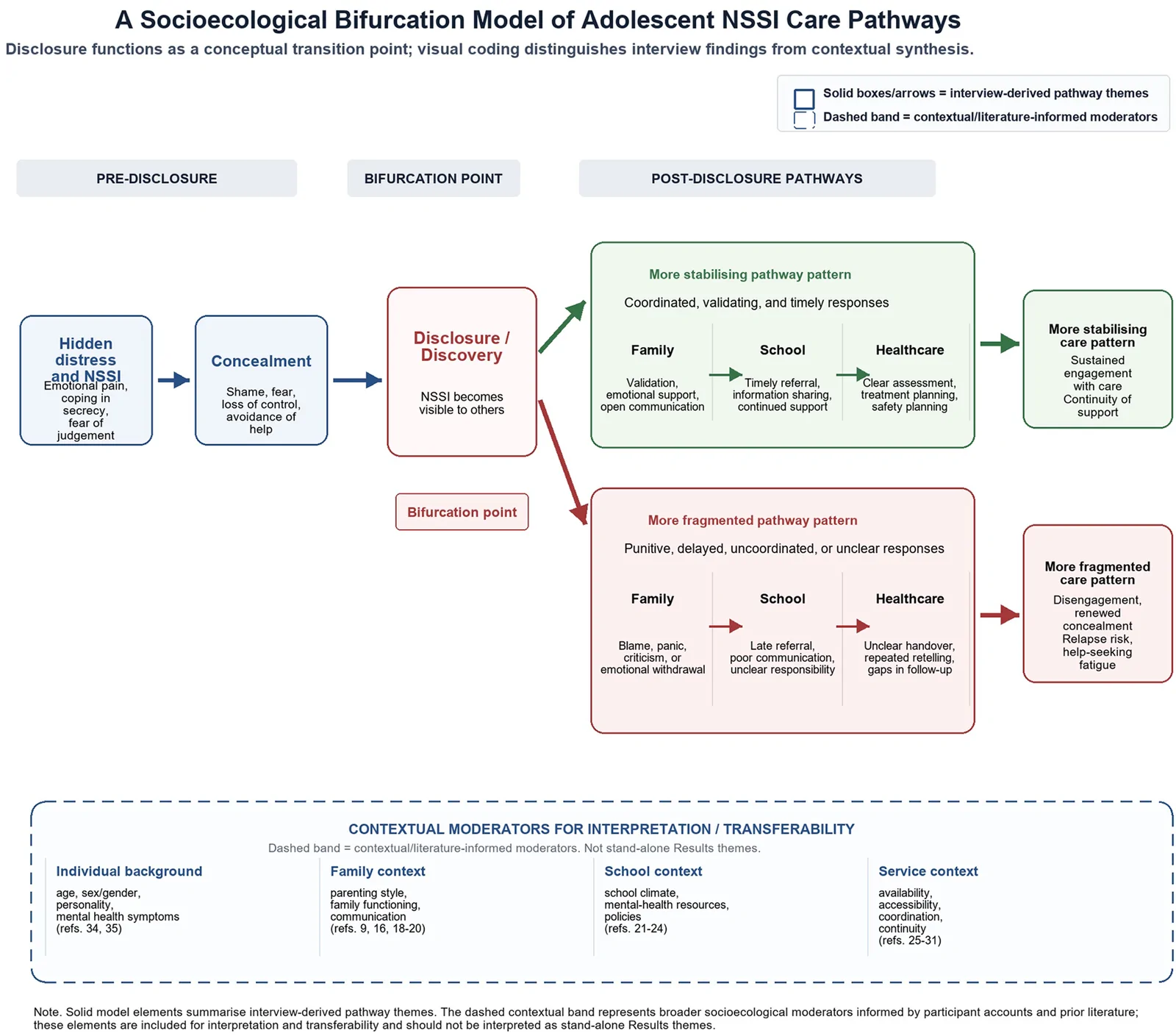
After disclosure, coordinated responses across family, school, and healthcare settings were described in relation to more stabilizing care patterns and greater continuity, whereas punitive, delayed, or fragmented responses were described in relation to renewed concealment, disengagement, and disrupted continuity of care. Solid pathway elements summarize empirical interview findings. The dashed contextual-influences band identifies broader socioecological moderators; individual background factors, school climate or policies, and service availability are included as literature-informed contextual considerations and should not be read as stand-alone Results themes (9, 10, 18, 20–33, 39, 40).

### Trustworthiness

Methodological rigor was supported through reflexive memo-writing, a transparent record of analytic decisions, iterative comparison between adolescent and caregiver accounts, and collaborative team discussions (14). These discussions were not used to adjudicate a single correct code or calculate coding reliability. Instead, preliminary interpretations were discussed to examine alternative explanations, identify assumptions linked to researchers’ clinical backgrounds, and strengthen the coherence of theme development in relation to both adolescent and caregiver perspectives.

### Ethical considerations

This study was approved by the Medical Ethics Committee of Shaoxing No. 7 Hospital (Approval No. 2025-031-01). Written informed consent was obtained from all caregivers, and written assent was obtained from all adolescent participants before participation. Participants were informed that participation was voluntary and that they could withdraw from the study at any time without consequences. To protect confidentiality, all identifying information was removed from transcripts before analysis, and de-identified participant codes were used throughout the manuscript.

A risk-management protocol was implemented for participants reporting recent self-harm, suicidal ideation, or acute psychological distress. Participants identified as having acute suicide risk during recruitment or interviews were referred for immediate clinical assessment and management according to standard clinical procedures and were not included in or continued with the interview process. Adolescents with a history of suicide attempts were excluded during eligibility assessment. Appropriate clinical support was arranged when necessary.

Before each interview, adolescents and caregivers were informed that their participation was voluntary and that they could pause or stop the interview at any time if they experienced distress. Adolescents were also informed that confidentiality would be maintained within ethical limits; however, if information disclosed during the interview indicated an immediate risk of serious harm, including imminent suicide risk or a situation requiring urgent protection, the research team would discuss appropriate safety actions and involve relevant caregivers and/or clinical professionals according to risk-management procedures.

During school-based interviews, interviews were conducted in private rooms, and researchers maintained communication pathways with the collaborating school mental health staff for situations requiring additional support. If significant emotional distress emerged during an interview, the interviewer would pause the discussion, provide emotional support, assess immediate safety needs, and facilitate referral to appropriate clinical resources when necessary. Information obtained from adolescent-only interviews was not routinely shared with caregivers; disclosure was limited to situations in which safety concerns required further intervention.

## Results

### Sample and pathway characteristics

The analytic sample included 20 adolescents and 20 caregivers (**Table 1**). Adolescents were aged 14–17 years, with a mean age of 15.6 years (SD 1.1). Thirteen adolescents were female and seven were male. The reported duration of NSSI had a median of 11.5 months (IQR 8.0-15.2; range 4-24). Discovery was mediated by parents or family members in six cases, peers or friends in five cases, school staff or counsellors in five cases, emergency or clinical contact in two cases, and other or mixed routes in two cases. School-based support or referral was the most common first service contact.

**Table 1:** Sample and care-pathway characteristics.

Characteristic	Category/statistic	n (%)
Age, years	Mean 15.6 (SD 1.1), range 14-17	
Sex	Female	13 (65%)
	Male	7 (35%)
School grade	Junior secondary, year 2	4 (20%)
	Junior secondary, year 3	5 (25%)
	Senior secondary, year 1	6 (30%)
	Senior secondary, year 2	5 (25%)
Reported NSSI duration	Median 11.5 months (IQR 8.0–15.2; range 4–24)	
Discovery pathway	Parent/family	6 (30%)
	Peer/friend	5 (25%)
	School staff/counsellor	5 (25%)
	Emergency/clinical contact	2 (10%)
	Other or mixed	2 (10%)
First service contact	School-based support/referral	10 (50%)
	Hospital or psychiatric service	7 (35%)
	Community counselling	1 (5%)
	Other	2 (10%)

Across dyads, NSSI care trajectories followed a six-stage pathway (**Tables 2**, **3**): accumulated distress, concealment and delayed disclosure, discovery and visibility transition, fragmented multi-system service entry, treatment engagement and family adaptation, and non-linear recovery with continuing care needs. The most important analytic finding was not only the sequence of stages, but the divergence of pathway patterns after NSSI became visible. When caregivers, schools, and clinical services were described as coordinated and validating, adolescents described greater continuity of engagement with care. When responses were described as punitive, delayed, or poorly coordinated, adolescents and caregivers described renewed concealment, repeated uncertainty, and difficulty sustaining follow-up.

**Table 2:** Six-stage socioecological care pathway model.

Stage	Core process	Analytic meaning
S1	Accumulated distress before disclosure	Academic, family, peer, and emotional stressors accumulated gradually before NSSI became visible or help-seeking began.
S2	Concealment and shame delayed disclosure	Shame, anticipated judgement, fear of parental reactions, and self-stigma reduced adolescents’ willingness to disclose distress or seek help.
S3	Discovery became a visibility transition	Detection by peers, family members, schools, or healthcare services transformed NSSI from a private experience into a shared care concern.
S4	Fragmented entry into multi-system services	Adolescents and caregivers entered school, outpatient, emergency, and psychiatric systems through often fragmented and poorly coordinated referral pathways.
S5	Treatment engagement and family adaptation	Continued engagement depended on therapeutic communication, family learning, and a gradual shift from blame to understanding and support.
S6	Non-linear recovery and continuity needs	Recovery required sustained follow-up, relapse-prevention planning, family communication, and cross-system continuity of care.

**Table 3:** Six-stage socioecological journey map of adolescent NSSI care.

Stage	Main experience	Touchpoints	Barriers	Facilitators	Level
Stage 1	Accumulated distress	Family, school, peers	Limited channels for disclosure; distress normalized	Peer awareness; teacher observation	Individual/family/school
Stage 2	Concealment and NSSI initiation	Self, online spaces, trusted friends	Shame, self-stigma, fear of parental blame	Trusted friends; non-judgemental response	Individual/peer/social
Stage 3	Discovery/disclosure	Friends, parents, class teachers, counsellors	Blame, denial, institutional anxiety	Stable caregiver response; timely school referral	Family/school
Stage 4	Help-seeking and service entry	School counsellor, psychiatry clinic, emergency department	Waiting, fragmented referral, unclear information	Clear pathway explanation; crisis assessment	School/health system
Stage 5	Treatment engagement	Psychiatrist, psychotherapist, nurse, caregiver	Weak alliance; focus on behavior without emotion	Supportive clinical communication; safety plan	Health system/family
Stage 6	Continuing care and relapse prevention	Outpatient follow-up, school, family, peers	Interrupted follow-up; old family patterns	Family learning; relapse-prevention plan; peer support	Individual/family/school/health

### Stage 1: accumulated psychological and contextual distress

Distress emerged gradually across academic, interpersonal, emotional, and family contexts before NSSI became visible to others. Adolescents described self-injury as arising from accumulated pressures rather than from a sudden isolated event. Sleep disturbance, emotional numbness, reduced concentration, and difficulty articulating distress often preceded help-seeking.

One adolescent said, “That period was mainly characterized by academic pressure and perfectionism. It did not happen suddenly; it felt as if everything accumulated gradually over time. At first I only slept poorly, but later I found it difficult to concentrate in class.” (A05, adolescent).

Another adolescent described prolonged emotional isolation following family disruption: “After my parents divorced, I often felt alone. I rarely talked about these feelings because I thought I should be able to cope on my own.” (A06, adolescent).

### Stage 2: concealment, self-stigma, and delayed disclosure

After NSSI began, adolescents often concealed both their distress and self-injury. Participants described concealment in relation to shame, fear of parental anger, anticipated blame, and uncertainty about whether others would understand. Rather than reflecting simple refusal of help, delayed disclosure reflected adolescents’ concern that disclosure could increase conflict or stigma.

One adolescent explained, “I hid it mainly because of shame. I was afraid my parents would be angry and I did not think anyone could really understand.” (A13, adolescent).

A second adolescent emphasized fear of social judgement: “I worried that people would see me differently if they found out. It felt safer to pretend everything was normal.” (A17, adolescent).

### Stage 3: discovery and visibility transition

Discovery marked a critical transition from hidden distress to shared concern. NSSI became visible through peers, parents, school staff, emergency contact, or clinical encounters. This transition was emotionally intense for both adolescents and caregivers. Caregivers often described fear, guilt, and confusion, while adolescents experienced exposure, panic, or loss of control over the situation.

A caregiver recalled, “When I first realized what had been happening, I felt frightened and confused. I kept asking myself whether I had missed the signs.” (P11, caregiver).

Another caregiver highlighted the role of school-based detection: “It was the school counsellor who contacted us. Without that call, we probably would not have recognized how much distress our child was carrying.” (P07, caregiver).

### Stage 4: fragmented entry into multi-system services

Following discovery, adolescents and families entered a fragmented network of school, outpatient, emergency, and psychiatric services. Participants described uncertainty about where to go, which service should take responsibility, and what the next step in care should be. School counsellors often served as initial contact points, but transitions between school and health systems were not always clearly coordinated.

An adolescent summarized this uncertainty: “The hardest part was not knowing what to do next. Different people gave different advice, and it was difficult to understand the process.” (A04, adolescent).

Participants also described uncertainty arising from multiple service transitions. One adolescent noted: “We moved between school staff, counsellors and hospital services. It felt like there was no clear pathway.” (A07, adolescent).

### Stage 5: treatment engagement and family adaptation

Treatment engagement was described in relation to the quality of communication, therapeutic alliance, and family adaptation. Adolescents described engagement as easier when professionals avoided blame and explained assessment, follow-up, and safety planning clearly. Caregivers also gradually shifted from interpreting NSSI as willfulness or defiance toward understanding it as a signal of distress requiring support.

One caregiver explained, “Over time I realized this was not simply willfulness. My child was trying to communicate emotional pain in a way that we had not understood.” (P14, caregiver).

Caregivers also described changes in their understanding of NSSI over time. One parent explained: “Learning more about self-injury changed the way we responded. We stopped focusing only on the behavior and paid more attention to emotions.” (P02, caregiver).

### Stage 6: non-linear recovery and continuity needs

Recovery was not described as a single endpoint after first contact. Adolescents and caregivers reported ongoing vulnerability during examinations, family conflict, social isolation, or emotional stress. Participants described continuing care as involving follow-up, relapse-prevention planning, family communication, school support, and a clear point of contact if risk re-emerged.

A caregiver stated, “It does not end after one clinic visit. We need a plan that can still be used if it happens again.” (P16, caregiver).

The importance of continuity was emphasized by another caregiver: “Ongoing communication between family, school and healthcare services was more important than any single appointment.” (P19, caregiver).

### Divergent pathway trajectories

Although the six stages were broadly shared across participants, pathway patterns diverged after NSSI became visible to others. Cases characterized by coordinated caregiver responses, timely school referral, and clear communication from health services tended to be described as more stabilizing. In these cases, adolescents described feeling understood, caregivers gradually shifted from shock to supportive engagement, and follow-up arrangements were clearly communicated. This combination of validation, coordination, and continuity appeared to facilitate sustained engagement with care.

In contrast, other cases were described as more fragmented, with delayed disclosure, caregiver panic or blame, unclear referral processes, repeated transitions between services, and uncertainty regarding responsibility for ongoing care. Adolescents in these trajectories described renewed concealment, reluctance to seek further help, and difficulty maintaining engagement after initial contact. These findings suggested that disclosure could be interpreted not only as a transition point but also as a possible bifurcation point, from which care trajectories appeared to move toward greater stabilization or fragmentation in relation to subsequent responses across family, school, and healthcare systems.

Together, these stages constitute a non-linear socioecological care pathway in which disclosure was associated with a redistribution of perceived responsibility across adolescent, family, school, and health-system levels. Stabilizing and fragmented pathways should be understood as interpretive patterns within participants’ accounts rather than fixed, deterministic, or mutually exclusive categories.

### Dyadic contrasts across key care transitions

Dyadic analysis indicated that adolescents and caregivers often assigned different meanings to similar care transitions. Across adolescent-caregiver dyads, adolescents tended to describe disclosure and service contact in terms of exposure, uncertainty, and concerns about judgement, whereas caregivers more frequently described these transitions in terms of fear, guilt, responsibility, and uncertainty about how to provide appropriate support. These differences reflected contrasting interpretations of shared care processes rather than disagreement about whether events had occurred.

Around the disclosure and discovery transition, adolescents commonly experienced increased visibility as emotionally threatening. One adolescent described concealing NSSI because of shame and fear that parents would respond negatively: “I hid it mainly because of shame. I was afraid my parents would be angry and I did not think anyone could really understand.” (A13, adolescent). In contrast, caregivers described discovery as a moment of fear and self-questioning. One caregiver explained: “When I first realized what had been happening, I felt frightened and confused. I kept asking myself whether I had missed the signs.” (P11, caregiver). Together, these accounts illustrate contrasting meanings that adolescents and caregivers assigned to disclosure and discovery across the dyadic dataset.

Differences were also evident during treatment engagement. Adolescents emphasized the importance of being understood and receiving explanations that reduced blame, whereas caregivers described a gradual process of learning to interpret NSSI as an expression of emotional distress rather than intentional misconduct. One caregiver stated: “Learning more about self-injury changed the way we responded. We stopped focusing only on the behavior and paid more attention to emotions.” (P02, caregiver). These contrasting perspectives suggest that continuity of care involved not only access to professional support but also the gradual development of shared understanding between adolescents and caregivers.

Taken together, these dyadic contrasts informed the interpretation of disclosure as a visibility-responsibility transition and contributed to the development of the socioecological care pathway model.

The tables and figures were developed to address complementary analytical purposes. **Table 2** defines the six-stage socioecological care pathway and summarizes the analytic meaning of each stage, whereas **Table 3** provides a detailed socioecological mapping of care touchpoints, barriers, facilitators, and system levels associated with each stage. **Figure 1** illustrates the temporal and relational experience of this pathway from adolescent and caregiver perspectives, highlighting dyadic differences in interpretation. **Figure 2** provides a conceptual synthesis of how disclosure or discovery may function as a bifurcation point associated with more stabilizing or more fragmented care trajectories.

### Illustrative within-dyad concordance and discordance

To further demonstrate how within-dyad analysis contributed to the identification of care trajectories, we examined paired adolescent-caregiver accounts from the same dyads around key transition points. Two illustrative cases are presented below, highlighting within-dyad discordance during NSSI disclosure and discovery and increasing concordance during treatment engagement and family adaptation. ((**Supplementary Table S2** provides additional paired quotations illustrating these within-dyad comparisons.))

In Dyad 13 (A13/P13), NSSI disclosure and discovery represented a critical transition from concealed distress to increased visibility within the family. However, adolescent and caregiver accounts revealed divergent interpretations of the same transition. The adolescent described concealment as closely related to shame and fear of negative reactions:”I hid it mainly because of shame. I was afraid my parents would be angry and I did not think anyone could really understand.” This account reflected the adolescent’s concerns about judgement and difficulty communicating emotional distress before disclosure. In contrast, the caregiver described discovery as an unexpected and distressing event characterized by fear and uncertainty:”When I first realized what had been happening, I felt frightened and confused. I kept asking myself whether I had missed the signs.”

Although both participants recognized the seriousness of the adolescent’s distress, they experienced this transition from different perspectives. The adolescent primarily experienced disclosure as exposure and vulnerability, whereas the caregiver experienced discovery as an alarming signal requiring understanding and support. This within-dyad discordance illustrates how the same transition could generate different emotional meanings among family members and may help explain variations in subsequent care trajectories.

In Dyad 2 (A02/P02), adolescent and caregiver accounts demonstrated increasing concordance during treatment engagement and family adaptation. The adolescent described distress as developing gradually in the context of accumulated pressures and family difficulties:”At that time it was mainly parent–child conflict and examination pressure. It did not happen suddenly; it felt like it had built up slowly.” Consistent with this account, the caregiver described a gradual shift in understanding NSSI, moving away from interpreting the behavior as intentional misconduct toward recognizing it as an expression of emotional distress:”Later I realized that this was not simply willfulness. It was a signal for help when my child did not know how to handle emotional pain.”

Although adolescent and caregiver experiences were initially framed differently, their accounts converged around the importance of understanding emotional distress and responding with greater support. Rather than focusing primarily on stopping the behavior, both perspectives became increasingly centered on communication, emotional needs, and reducing blame. This within-dyad concordance reflected a stabilizing trajectory characterized by improved family adaptation and continued engagement with care.

Together, these paired examples demonstrate that within-dyad analysis involved examining how members of the same family interpreted and responded to shared transition points, rather than simply comparing adolescent and caregiver narratives separately. Discordance was evident during disclosure and discovery, when adolescents and caregivers attributed different meanings to the same event, whereas concordance emerged during treatment engagement as families developed shared understandings of distress and support needs.

### Dyadic journey map

The dyadic journey map is presented in **Figure 1**.

### A socioecological bifurcation model of adolescent NSSI care pathways

The socioecological bifurcation model is presented in **Figure 2** as an interpretive conceptual model rather than a causal model. In the revised figure, solid pathway elements denote interview-derived themes, while the dashed contextual band denotes contextual and literature-informed moderators rather than additional Results themes.

## Discussion

### Principal findings

This dyadic qualitative study conceptualizes adolescent non-suicidal self-injury (NSSI) as a socioecological care pathway rather than a linear individual help-seeking process. The central finding is that disclosure or discovery of NSSI was described as a critical transition point, through which concealed distress became a shared care concern and perceived responsibility was redistributed across adolescents, caregivers, schools, and health services. After this transition, care pathways were described as diverging. Coordinated and validating responses appeared to support continued engagement and continuity of care, whereas delayed, punitive, or fragmented responses were associated with renewed concealment, uncertainty, and pathway instability.

These findings suggest that continuity of care after adolescent NSSI is not determined solely by service access or individual willingness to seek help. Rather, participants’ accounts indicated that continuity was associated with interactions between adolescents’ willingness to remain visible within care networks and the capacity of family, school, and healthcare systems to provide coherent and non-judgemental responses. The bifurcation model should therefore be understood as an interpretive conceptual model of pathway patterning, not as a causal model predicting fixed outcomes.

### Comparison with existing literature

The findings extend existing literature on adolescent NSSI, while also drawing on relevant evidence from the broader adolescent self-harm literature where appropriate, by moving beyond intrapersonal explanations of self-injury, such as affect regulation and self-punishment, to emphasize the relational and organizational conditions under which help-seeking may become possible. While stigma, shame, and limited mental health literacy remain important barriers to disclosure, participants’ accounts suggested that these barriers were not only individual psychological states. They were also described in relation to anticipated parental reactions, school responses, referral clarity, and the perceived reliability of clinical services (15–26).

The dyadic design further shows that adolescents and caregivers may experience the same care transition differently. Adolescents tended to emphasize shame, exposure, fear of judgement, and loss of control after disclosure, whereas caregivers described shock, guilt, uncertainty, and difficulty knowing how to respond. This mismatch offers one interpretation of why disclosure may not automatically be followed by stable care. In participants’ accounts, caregivers’ fear and confusion were sometimes experienced by adolescents as blame or rejection, which appeared to be associated with renewed concealment and reduced engagement with care (9, 16–22).

### Theoretical contribution

This study contributes to the adolescent NSSI literature in three ways. First, it shifts the conceptual focus from individual help-seeking behavior to care-pathway development. Existing research has largely examined NSSI disclosure as a discrete event or decision influenced by stigma, emotional distress, and help-seeking attitudes. In contrast, the present study conceptualizes disclosure as a dynamic transition within a broader socioecological pathway. The findings suggest that disclosure does not represent the endpoint of concealment but rather the beginning of a new phase of care navigation involving multiple interacting systems (15–19).

Second, this study introduces the concept of disclosure as a pathway bifurcation point. Participants’ accounts suggested that the same disclosure event could be followed by markedly different trajectories in relation to subsequent responses from caregivers, schools, and healthcare services. Coordinated and validating responses were associated with engagement, continuity, and recovery-oriented support, whereas fragmented or punitive responses were associated with concealment, uncertainty, and disengagement. This finding extends existing disclosure research by suggesting that post-disclosure system responses are important for understanding how different care pathways are experienced (15–26).

Third, the study advances a socioecological understanding of continuity of care. Existing studies often conceptualize continuity as a service-delivery outcome or a characteristic of healthcare systems. The present findings suggest that continuity may be understood as emerging through the interaction of individual, family, school, and healthcare processes over time. From this perspective, continuity of care is interpreted as an emergent property of system coordination rather than solely as an outcome of service access or treatment adherence (27–33).

Taken together, these contributions provide a process-oriented framework for understanding how adolescent NSSI care pathways may evolve following disclosure and how similar disclosure experiences may be associated with either more stabilizing or more fragmented care patterns.

### Family, school, and service-system mechanisms

Family response was described as an important relational process associated with pathway stability. Initial caregiver reactions of panic, guilt, or blame appeared to intensify adolescent distress and reduce willingness to disclose further difficulties. However, when caregivers shifted towards emotional validation, safety-oriented communication, and practical support, participants described the family as a more stabilizing component of the care pathway.

This interpretation is consistent with findings from the broader adolescent self-harm literature, which has highlighted the importance of caregiver responses after self-harm disclosure. Although these studies are not exclusively focused on NSSI, they provide relevant contextual support for understanding caregiver involvement in adolescent NSSI care pathways (9, 20–22).

Schools also played a central role as early detection and referral points. In many cases, school staff or counsellors were among the first formal contacts after NSSI became visible. Timely, non-judgemental school responses appeared to facilitate access to professional care, whereas unclear school roles or weak referral pathways were described as increasing uncertainty for both adolescents and caregivers. These findings support the need to strengthen school-based identification, referral, and follow-up procedures as part of an integrated adolescent mental health care continuum (22–26).

Service fragmentation was described as the major system-level barrier. Participants described movement between school counsellors, emergency departments, outpatient clinics, and psychiatric services without clear handover or continuity planning. This fragmentation appeared to increase emotional burden and reduce confidence in the care system. From a socioecological perspective, pathway instability was interpreted as reflecting poor alignment between system levels rather than failure at a single point of care (27–33).

### Contextual influences in China

Participants’ accounts situated NSSI care pathways within educational, family, and school contexts that are particularly salient in the Chinese setting. Academic pressure was frequently described by adolescents as part of the distress preceding NSSI, while caregivers’ accounts highlighted family responsibility, uncertainty, and the challenges of responding after disclosure. Schools also appeared repeatedly in participants’ narratives as important points of detection, referral, and communication between families and formal mental health services.

These findings should be interpreted within the specific educational and service context in which the study was conducted. Broader features of the Chinese context, including intense academic competition, family-centered expectations, and variation in school-based mental health resources (34–36), may provide important background for understanding these experiences, but were not examined as independent themes in the present study. Accordingly, the proposed model should be understood as a socioecological interpretation grounded in participants’ accounts and situated within a specific local cultural and service context.

### Implications for practice

The findings have several implications for adolescent mental health care. First, post-disclosure responses should prioritize emotional safety, non-judgemental communication, and clear explanation of next steps (15–26). Second, caregivers require structured support to move from shock and self-blame towards effective communication and relapse-prevention planning (20, 21, 37, 38). Third, schools need explicit protocols for identifying NSSI, communicating with families, referring to health services, and maintaining appropriate follow-up (22–26). Fourth, clinical services should provide adolescents and caregivers with a clear care map, including assessment, safety planning, follow-up arrangements, and points of contact if risk re-emerges (27–33, 39).

Together, these strategies may help reduce repeated uncertainty and improve continuity across family, school, and healthcare systems. Rather than treating disclosure as the endpoint of help-seeking, services should treat it as the beginning of coordinated pathway management (15–33, 37–39).

### Strengths and limitations

A key strength of this study is its dyadic design, incorporating both adolescent and caregiver perspectives. This allowed the analysis to identify not only individual experiences but also mismatches between adolescents’ and caregivers’ interpretations of disclosure, service entry, and continuing care. The use of socioecological journey mapping also enabled integration of temporal progression and multi-level system interactions within a single analytical framework.

Several limitations should be considered. The sample was recruited from one city in China and may not represent adolescents in other regions or service contexts. The sample size was appropriate for qualitative depth but not intended for statistical generalization. Retrospective interviews may be affected by recall bias, especially regarding the timing and sequence of early disclosure events. The study may also under-represent adolescents whose NSSI remained fully concealed or who never accessed formal services. In addition, requiring participation from a caregiver involved in the adolescent’s daily care may have preferentially included adolescents with available and engaged caregivers who were willing and able to participate in qualitative interviews, and may therefore limit representation of adolescents with limited caregiver involvement or reduced access to family support. Recruitment through clinical and school-based pathways may also have influenced the types of care trajectories represented, as participants had already reached at least one point of visibility within formal or semi-formal support systems. Furthermore, because caregiver relationship characteristics were not systematically collected, we were unable to examine whether different caregiver roles (e.g., mothers, fathers, or other caregivers) shaped adolescents’ experiences of disclosure, family response, or care trajectories. Future studies should incorporate caregiver role characteristics to further explore potential relational differences. Finally, although caregiver perspectives were included, the study did not directly include teachers, school counsellors, or clinicians as participants; future research could incorporate these perspectives to further examine cross-system coordination.

### Conclusion

Adolescent NSSI care is best understood as a non-linear socioecological pathway associated with disclosure, system responses, and cross-level coordination. Disclosure or discovery is not simply an endpoint of concealment but was interpreted in this study as a turning point after which care could move toward more stabilizing or more fragmented patterns. These pathways should not be understood as fixed or mutually exclusive. Improving continuity of care may require coordinated responses across families, schools, and healthcare services, with particular attention to disclosure safety, referral clarity, caregiver support, and relapse-prevention planning.

## Data Availability

The original contributions presented in the study are included in the article/**Supplementary Material**. Further inquiries can be directed to the corresponding author/s.
