# Supplementary material for: Care pathways following disclosure of adolescent non-suicidal self-injury: a dyadic qualitative study
Source: Front Psychiatry. 2026 Sep 30;17:1934223. doi: 10.3389/fpsyt.2026.1934223 (PMC13661548; doi:10.3389/fpsyt.2026.1934223)
Supplement: Supplementary file 1 [file Table1.docx]

**Supplementary Table S1. Operationalized Codebook**

The codebook maps 25 open codes to socioecological levels and journey stages. Because some codes represented evolving processes across the care trajectory, certain codes were assigned to more than one journey stage. Theme assignment reflected the dominant interpretive role of each code within the corresponding theme rather than a one-to-one correspondence between codes and stages.

| **Code ID** | **Code** | **Socioecological level** | **Journey stage** | **Operational definition** |
| --- | --- | --- | --- | --- |
| C01 | Academic pressure | Individual/school | S1 | Sustained tension related to academic workload, examinations, grades, and performance evaluation. |
| C02 | Family conflict | Family | S1/S5 | Parent–adolescent conflict, disrupted communication, family tension, or old interaction patterns. |
| C03 | Peer rejection | Peer/school | S1/S3 | Peer exclusion, isolation, ridicule, or conflict that contributed to distress or detection. |
| C04 | Emotional numbness | Individual | S1 | Numbness, emptiness, difficulty naming emotions, or reduced ability to communicate distress. |
| C05 | Self-punishment | Individual | S2 | Self-injury interpreted as punishment of the self or a way of reducing guilt. |
| C06 | Shame and concealment | Individual/family | S2 | Shame, hiding injuries, hiding distress, or avoiding others' awareness of NSSI. |
| C07 | Fear of parental reaction | Family | S2/S3 | Concern that parents would blame, misunderstand, become angry, or emotionally collapse. |
| C08 | Peer detection | Peer | S3 | Friends noticed injuries, mood changes, or unusual behaviour and became part of disclosure. |
| C09 | Parent discovery | Family | S3 | Caregivers discovered NSSI through clothing, injuries, mood changes, or direct observation. |
| C10 | Teacher/counsellor recognition | School | S3/S4 | Teachers or school counsellors recognised possible risk and initiated contact or referral. |
| C11 | Delayed help-seeking | Individual/family/system | S2/S4 | Time gap between first NSSI and formal help-seeking due to concealment, stigma, or uncertainty. |
| C12 | School as first contact | School | S4 | School counsellors or class teachers served as the first formal point of mental health contact. |
| C13 | Emergency pathway | Health system | S4 | Entry into care through emergency department or urgent clinical assessment. |
| C14 | Outpatient psychiatry | Health system | S4/S5 | Entry into or ongoing care through child psychiatry or psychological outpatient clinics. |
| C15 | Fragmented referral | Health system | S4 | Multiple referrals, unclear handovers, inconsistent information, or poor coordination between services. |
| C16 | Long waiting time | Health system | S4 | Waiting for appointments, emergency assessment, or inpatient beds. |
| C17 | Supportive clinician | Health system | S5 | Clinicians listened, explained assessment/follow-up, and communicated without blame. |
| C18 | Invalidating response | Family/school/health | S3/S5 | Responses that framed NSSI as attention-seeking, wilful, exaggerated, or morally wrong. |
| C19 | Family learning | Family | S5/S6 | Caregivers gradually understood NSSI as distress communication and adjusted their response. |
| C20 | Relapse trigger | Individual/school/family | S6 | Examinations, family conflict, interpersonal stress, or isolation reactivated urges or risk. |
| C21 | Peer support | Peer | S3/S6 | Friends accompanied, encouraged help-seeking, or helped adolescents disclose safely. |
| C22 | Need for continuity | Health system | S6 | Need for follow-up after outpatient or emergency contact and continuity after initial service entry. |
| C23 | Unmet informational needs | Family/health system | S4/S5 | Caregivers did not know whom to contact, how to respond, or how to prevent recurrence. |
| C24 | Recovery as nonlinear | Individual/family | S6 | Recovery was described as recurrent, cyclical, and not a single endpoint after first treatment. |
| C25 | Stigma | Social | S2/S4 | Stigma surrounding psychiatry, psychological problems, self-injury, and professional help-seeking. |

# Theme matrix

| **Theme ID** | **Theme / stage** | **Analytic explanation** | **Included codes** | **Stage** |
| --- | --- | --- | --- | --- |
| T1 | Accumulated distress before disclosure | Academic, family, peer, and emotional stressors accumulated before NSSI became visible or help-seeking began. | C01, C02, C03, C04 | S1 |
| T2 | Concealment and shame delayed disclosure | Shame, self-directed negative emotions, fear of parental blame, self-stigma, and delayed help-seeking reduced disclosure safety. | C05, C06, C07, C11, C25 | S2 |
| T3 | Discovery became a visibility transition | Discovery by peers, parents, school staff, or clinicians shifted NSSI from a private experience to a shared care concern. | C08, C09, C10, C18, C21 | S3 |
| T4 | Fragmented entry into multi-system services | Families moved between school, emergency, outpatient, and psychiatric systems without clear handover or pathway explanation. | C12, C13, C14, C15, C16, C23 | S4 |
| T5 | Treatment engagement and family adaptation | Engagement improved with supportive clinical communication, psychoeducation, and caregiver learning. | C17, C18, C19, C23 | S5 |
| T6 | Non-linear recovery and continuity needs | Recovery required follow-up, relapse-prevention planning, family communication, and cross-system continuity. | C20, C21, C22, C24 | S6 |
