# Supplementary material for: Care pathways following disclosure of adolescent non-suicidal self-injury: a dyadic qualitative study
Source: Front Psychiatry. 2026 Sep 30;17:1934223. doi: 10.3389/fpsyt.2026.1934223 (PMC13661548; doi:10.3389/fpsyt.2026.1934223)
Supplement: Supplementary file 2 [file Table2.docx]

# Supplementary Table S2. Illustrative Quotations Supporting Theme Development

Note: Quotations are presented as translated excerpts from the original Mandarin interviews.

## Theme 1. Accumulated Psychological and Contextual Distress

**A05 (Adolescent):** That period was mainly characterised by academic pressure and perfectionism. It did not happen suddenly; it felt as if everything accumulated gradually over time. At first I only slept poorly, but later I found it difficult to concentrate in class.

**A06 (Adolescent):** After my parents divorced, I often felt alone. I rarely talked about these feelings because I thought I should be able to cope on my own.

**A13 (Adolescent):** Conflicts on social media made me feel embarrassed and ashamed. I kept replaying things in my mind and gradually stopped telling other people how I felt.

**A16 (Adolescent):** Examinations and worries about the future were always there. Over time I felt exhausted and unable to manage the pressure.

## Theme 2. Concealment, Shame and Delayed Disclosure

**A13 (Adolescent):** I hid it mainly because of shame. I was afraid my parents would be angry and I did not think anyone could really understand.

**A17 (Adolescent):** I worried that people would see me differently if they found out. It felt safer to pretend everything was normal.

**A20 (Adolescent):** Even when someone asked if I was okay, I usually said I was fine because I did not know how to explain what was happening.

**A04 (Adolescent):** I kept everything to myself for a long time. I thought talking about it would only create more problems.

## Theme 3. Discovery and Visibility Transition

**P11 (Caregiver):** When I first realised what had been happening, I felt frightened and confused. I kept asking myself whether I had missed the signs.

**P04 (Caregiver):** The emergency visit made us realise the seriousness of the situation. Before that, we did not fully understand what our child was experiencing.

**P07 (Caregiver):** It was the school counsellor who contacted us. Without that call, we probably would not have recognised how much distress our child was carrying.

**P20 (Caregiver):** When the psychiatrist explained self-injury to us, we understood that this was emotional suffering rather than deliberate misbehaviour.

## Theme 4. Fragmented Entry into Services

**A04 (Adolescent):** The hardest part was not knowing what to do next. Different people gave different advice, and it was difficult to understand the process.

**A07 (Adolescent):** We moved between school staff, counsellors and hospital services. It felt like there was no clear pathway.

**A10 (Adolescent):** There was a lot of waiting and uncertainty before we reached the right service.

**A12 (Adolescent):** Each place seemed responsible for only one part of the problem. Nobody explained how everything connected together.

## Theme 5. Treatment Engagement and Family Adaptation

**P14 (Caregiver):** Over time I realised this was not simply wilfulness. My child was trying to communicate emotional pain in a way that we had not understood.

**P02 (Caregiver):** Learning more about self-injury changed the way we responded. We stopped focusing only on the behaviour and paid more attention to emotions.

**P10 (Caregiver):** The therapist helped us communicate differently. Criticism only increased tension, whereas listening made a difference.

**P18 (Caregiver):** As a family we gradually became more supportive. Understanding came before improvement.

## Theme 6. Non-linear Recovery and Continuity Needs

**P16 (Caregiver):** Recovery was not a straight line. There were periods of improvement and periods when stress brought the difficulties back.

**P01 (Caregiver):** We needed a plan for what to do when problems returned, not just support during the crisis.

**P11 (Caregiver):** Follow-up appointments helped us feel that someone was still accompanying us through the process.

**P19 (Caregiver):** Ongoing communication between family, school and healthcare services was more important than any single appointment.

**Additional within-dyad illustrative quotations**

Note:

The following paired quotations are presented to further illustrate within-dyad comparisons. Quotations are selected from the same adolescent-caregiver dyads and organised by key care transitions to demonstrate concordance and discordance.

| **Dyad** | **Transition point** | **Participant** | **Illustrative quotation** | **Within-dyad interpretation** |
| --- | --- | --- | --- | --- |
| Dyad 13 (A13/P13) | NSSI disclosure and discovery | Adolescent (A13) | “I hid it mainly because of shame. I was afraid my parents would be angry and I did not think anyone could really understand.” | The adolescent account reflected concealment, shame, fear of judgement, and difficulty communicating emotional distress before disclosure. |
| Dyad 13 (A13/P13) | NSSI disclosure and discovery | Caregiver (P13) | “When I first realised what had been happening, I felt frightened and confused. I kept asking myself whether I had missed the signs.” | The caregiver account reflected fear, uncertainty, and recognition of previously hidden distress after discovery. |
| Dyad 2 (A02/P02) | Treatment engagement and family adaptation | Adolescent (A02) | “At that time it was mainly parent–child conflict and examination pressure. It did not happen suddenly; it felt like it had built up slowly.” | The adolescent account reflected accumulated distress and the need for understanding during the care process. |
| Dyad 2 (A02/P02) | Treatment engagement and family adaptation | Caregiver (P02) | “Later I realised that this was not simply wilfulness. It was a signal for help when my child did not know how to handle emotional pain.” | The caregiver account reflected a shift from behavioural interpretation toward understanding emotional distress. |
