# Supplementary material for: Care pathways following disclosure of adolescent non-suicidal self-injury: a dyadic qualitative study
Source: Front Psychiatry. 2026 Sep 30;17:1934223. doi: 10.3389/fpsyt.2026.1934223 (PMC13661548; doi:10.3389/fpsyt.2026.1934223)
Supplement: Supplementary file 3 [file Table3.docx]

**Supplementary File: Semi-structured Interview Guide**

Manuscript: Care pathways following disclosure of adolescent non-suicidal self-injury: A dyadic qualitative study

The guide was designed for separate adolescent and caregiver interviews. Interviewers used open-ended prompts and follow-up questions, avoiding detailed discussion of self-injury methods and prioritising participant safety.

# A. Adolescent interview guide

1. Can you tell me about the period before NSSI first became part of your life? What was happening at school, at home, with peers, and emotionally?
2. Before anyone else knew, did you try to tell anyone that you were struggling? What made it easier or harder to say something?
3. How did you understand NSSI at that time? What did it mean to you emotionally?
4. Did you hide the distress or NSSI from others? What were you worried might happen if people found out?
5. How was the NSSI discovered or disclosed? Who became involved first?
6. What happened after discovery? How did your family, school, peers, or clinicians respond?
7. How did you enter mental health services? Who explained the next steps, and what felt unclear?
8. What parts of school, outpatient, emergency, or psychiatric care helped? What parts felt difficult or fragmented?
9. How did treatment or counselling affect you? What helped you trust or distrust professionals?
10. What does recovery mean now? What kinds of follow-up, safety planning, family support, school support, or relapse-prevention help do you need?

# B. Caregiver interview guide

11. Can you describe what you noticed before you knew about the NSSI?
12. How did you discover or learn about the NSSI? What was your immediate reaction?
13. What did you understand NSSI to mean at first, and how did that understanding change over time?
14. What did you do after discovery? Whom did you contact first: school, hospital, community counselling, emergency service, or another source?
15. What was unclear or difficult when navigating school and healthcare services?
16. How did school staff or counsellors respond? What helped or hindered referral?
17. How did healthcare professionals communicate risk assessment, NSSI, suicidal intent, treatment, and follow-up?
18. What support did you need as a caregiver?
19. What changes occurred in family communication after discovery and treatment?
20. What kind of continuing care, relapse-prevention plan, school coordination, and family guidance would help now?

# C. Safety and closing prompts

21. Is there anything important about your care journey that we have not discussed?
22. How are you feeling after the interview?
23. Do you need immediate support or would you like us to connect you with the clinical contact according to the risk-management protocol?

Interviewer note: If recent self-harm, suicidal ideation, or acute distress is reported, pause the interview and activate the approved risk-management protocol.
