# Supplementary material for: Care pathways following disclosure of adolescent non-suicidal self-injury: a dyadic qualitative study
Source: Front Psychiatry. 2026 Sep 30;17:1934223. doi: 10.3389/fpsyt.2026.1934223 (PMC13661548; doi:10.3389/fpsyt.2026.1934223)
Supplement: Supplementary file 4 [file Table4.docx]

Supplementary File: Research Team Reflexivity Statement

Manuscript: Care pathways following disclosure of adolescent non-suicidal self-injury: A dyadic qualitative study

# Research team and positionality

Interviews were conducted by two trained researchers with prior experience in adolescent mental health qualitative research: a psychiatric nurse researcher and a child psychiatrist. Both researchers were familiar with clinical and school-based adolescent mental health contexts but were not in a prior therapeutic, teaching or supervisory relationship with participants.

# Relationship with participants

Adolescents and caregivers were recruited through adolescent psychiatric outpatient services and collaborating secondary schools. Interviews were conducted separately to reduce family influence on participant accounts. No interviewer had a prior therapeutic, teaching or supervisory relationship with the participants.

# Reflexive stance

The analytic team treated NSSI not only as an individual symptom but also as a care pathway shaped by family response, school detection, and health-system coordination. To reduce premature interpretation, analysts compared adolescent and caregiver accounts iteratively and used reflexive memos to record assumptions about stigma, family responsibility, and service fragmentation.

# Managing analytic influence

During coding, the team distinguished participant descriptions from analytic interpretation. Meaning units were first organized chronologically, then mapped to socioecological levels. Differences in interpretation were explored through reflexive discussion, with reference to the audit trail, coding records, and journey-mapping matrices to support analytic transparency and refinement of interpretations.

# Transparency statement

The research team maintained reflexive memos throughout data collection and analysis to document assumptions, emotional responses, and analytic decisions. The team paid particular attention to the risk of over-pathologising adolescents' accounts or over-attributing responsibility to caregivers. Adolescent and caregiver narratives were compared iteratively to preserve dyadic differences while constructing the shared care pathway model.
