# Supplementary material for: Care pathways following disclosure of adolescent non-suicidal self-injury: a dyadic qualitative study
Source: Front Psychiatry. 2026 Sep 30;17:1934223. doi: 10.3389/fpsyt.2026.1934223 (PMC13661548; doi:10.3389/fpsyt.2026.1934223)
Supplement: Supplementary file 5 [file Table5.docx]

# COREQ Checklist for Qualitative Manuscript

Manuscript: Care pathways following disclosure of adolescent non-suicidal self-injury: A dyadic qualitative study

| COREQ item | Status | Information reported | Manuscript location |
| --- | --- | --- | --- |
| 1. Interviewer/facilitator | Reported | Two trained researchers conducted separate interviews with adolescents and caregivers. | Methods: Research team and reflexivity |
| 2. Credentials | Not reported | Specific academic credentials or degrees of the interviewers were not reported. | Not reported |
| 3. Occupation | Reported | Interviewers were a psychiatric nurse researcher and a child psychiatrist. | Methods: Research team and reflexivity; Supplementary reflexivity statement |
| 4. Gender | Not reported | Gender of the interviewers was not reported. | Not reported |
| 5. Experience and training | Partially reported | Interviewers had prior experience in adolescent mental health qualitative research; specific training details were not reported. | Methods: Research team and reflexivity; Supplementary reflexivity statement |
| 6. Relationship established | Reported | No prior therapeutic, teaching, or supervisory relationship existed between researchers and participants. | Methods: Research team and reflexivity; Supplementary reflexivity statement |
| 7. Participant knowledge of interviewer | Not reported | Participants' knowledge of the interviewers' backgrounds, personal goals, or reasons for conducting the research was not reported. | Not reported |
| 8. Interviewer characteristics | Reported | Reflexive journals and memos documented assumptions, clinical backgrounds, positionality, emotional responses, and analytic decisions; regular team discussions examined potential interpretive influence. | Methods: Research team and reflexivity; Supplementary reflexivity statement |
| 9. Methodological orientation and theory | Reported | The study used a contextualist epistemological orientation; reflexive thematic analysis was combined with socioecological journey mapping. | Methods: Research team and reflexivity; Data analysis |
| 10. Sampling | Reported | Purposive sampling recruited adolescents with documented NSSI and one corresponding caregiver, with variation in age, sex, school grade, and help-seeking experience. | Methods: Participants and recruitment |
| 11. Method of approach | Reported | Potential participants were identified by treating psychiatrists or school mental-health staff and subsequently approached by the research team. | Methods: Participants and recruitment |
| 12. Sample size | Reported | 20 adolescents and 20 corresponding caregivers. | Results: Table 1 |
| 13. Non-participation | Partially reported | Eight eligible families declined participation because of time constraints or reluctance to discuss self-injury experiences. | Methods: Participants and recruitment |
| 14. Setting of data collection | Reported | Outpatient consultation rooms and school counselling rooms. | Methods: Data collection |
| 15. Presence of non-participants | Partially reported | Adolescents and caregivers were interviewed separately; the presence of non-participants was not explicitly reported. | Methods: Data collection |
| 16. Description of sample | Partially reported | Adolescent age, sex, grade, NSSI duration, discovery pathway, and first service contact are summarized; caregiver demographic and relationship characteristics were not systematically collected. | Methods: Participants and recruitment; Results: Table 1 |
| 17. Interview guide | Reported / supplementary | The semi-structured interview guide is provided as supplementary material. | Supplementary File: Semi-structured Interview Guide |
| 18. Repeat interviews | Reported | No repeat interviews were conducted. | Methods: Data collection |
| 19. Audio/visual recording | Reported | All interviews were audio-recorded and transcribed verbatim. | Methods: Data collection |
| 20. Field notes | Reported | Field notes were completed after each interview; analytic memos and thematic-development logs were also maintained. | Methods: Data collection; Research team and reflexivity |
| 21. Duration | Reported | Each interview lasted approximately 46-58 minutes. | Methods: Data collection |
| 22. Data saturation | Partially reported | Interpretive sufficiency, rather than conventional coding saturation, was considered reached when additional interviews no longer substantially elaborated conceptual categories or pathway transitions across three consecutive interviews. | Methods: Data analysis |
| 23. Transcripts returned | Reported | Transcripts were not routinely returned to participants because of the sensitive nature of the topic and concerns regarding participant burden. | Methods: Data collection |
| 24. Number of data coders | Reported | One researcher (JLF) initially coded all transcripts. Other team members participated in reflexive discussions of emerging codes and themes, providing alternative perspectives and helping refine interpretations. | Methods: Data analysis |
| 25. Description of coding tree | Reported / supplementary | An operationalised codebook is provided in Supplementary Table S1. | Supplementary Table S1: Operationalised Codebook |
| 26. Derivation of themes | Reported | Themes were derived inductively from meaning units and coding records, then synthesised into six empirically grounded stages. | Methods: Data analysis |
| 27. Software | Reported | Microsoft Excel. | Methods: Data analysis |
| 28. Participant checking | Not reported | The manuscript states that transcripts were not routinely returned, but it does not explicitly report whether participants were asked to comment on the findings. | Not reported; related transcript-return information in Methods: Data collection |
| 29. Quotations presented | Reported | Representative quotations from adolescents and caregivers are presented in the Results and Supplementary Table S2. | Results; Supplementary Table S2: Illustrative Quotations Supporting Theme Development |
| 30. Data and findings consistent | Reported | Findings are supported by representative quotations and aligned with the reported Tables and Figures. | Results; Tables 1-3; Figures 1-2 |
| 31. Clarity of major themes | Reported | Six major stages/themes are clearly described. | Results |
| 32. Clarity of minor themes | Reported | Barriers, facilitators, touchpoints, and socioecological levels are summarised. | Results; Table 3; Figure 1 |
